# Two alternative recessive quantitative trait loci influence resistance to spring black stem and leaf spot in *Medicago truncatula*

**DOI:** 10.1186/1471-2229-8-30

**Published:** 2008-03-26

**Authors:** Lars G Kamphuis, Judith Lichtenzveig, Richard P Oliver, Simon R Ellwood

**Affiliations:** 1 Australian Centre for Necrotrophic Fungal Pathogens, State Agricultural Biotechnology Centre, Murdoch University, Perth 6150, Western Australia, Australia; 2 Commonwealth Scientific and Industrial Research Organisation, Plant Industry, Private Bag No. 5, Wembley 6913, Western Australia, Australia

## Abstract

**Background:**

Knowledge of the genetic basis of plant resistance to necrotrophic pathogens is incomplete and has been characterised in relatively few pathosystems. In this study, the cytology and genetics of resistance to spring black stem and leaf spot caused by *Phoma medicaginis*, an economically important necrotrophic pathogen of *Medicago *spp., was examined in the model legume *M. truncatula*.

**Results:**

Macroscopically, the resistant response of accession SA27063 was characterised by small, hypersensitive-like spots following inoculation while the susceptible interaction with accessions A17 and SA3054 showed necrotic lesions and spreading chlorosis. No unique cytological differences were observed during early infection (<48 h) between the resistant and susceptible genotypes, except pathogen growth was restricted to one or a few host cells in SA27063. In both interactions reactive oxygen intermediates and phenolic compounds were produced, and cell death occurred. Two F_2 _populations segregating for resistance to spring black stem and leaf spot were established between SA27063 and the two susceptible accessions, A17 and SA3054. The cross between SA27063 and A17 represented a wider cross than between SA27063 and SA3054, as evidenced by higher genetic polymorphism, reduced fertility and aberrant phenotypes of F_2 _progeny. In the SA27063 × A17 F_2 _population a highly significant quantitative trait locus (QTL, LOD = 7.37; *P *< 0.00001) named resistance to the necrotroph *Phoma medicaginis *one (*rnpm1*) genetically mapped to the top arm of linkage group 4 (LG4). *rnpm1 *explained 33.6% of the phenotypic variance in the population's response to infection depicted on a 1–5 scale and was tightly linked to marker AW256637. A second highly significant QTL (LOD = 6.77; *P *< 0.00001), *rnpm2*, was located on the lower arm of LG8 in the SA27063 × SA3054 map. *rnpm2 *explained 29.6% of the phenotypic variance and was fine mapped to a 0.8 cM interval between markers h2_16a6a and h2_21h11d. *rnpm1 *is tightly linked to a cluster of Toll/Interleukin1 receptor-nucleotide binding site-leucine-rich repeat (TIR-NBS-LRR) genes and disease resistance protein-like genes, while no resistance gene analogues (RGAs) are apparent in the genomic sequence of the reference accession A17 at the *rnpm2 *locus.

**Conclusion:**

The induction of defence responses and cell death in the susceptible interaction following infection by *P. medicaginis *suggested this pathogen is not negatively affected by these responses and may promote them. A QTL for resistance was revealed in each of two populations derived from crosses between a resistant accession and two different susceptible accessions. Both loci are recessive in nature, and the simplest explanation for the existence of two separate QTLs is the occurrence of host genotype-specific susceptibility loci that may interact with undetermined *P. medicaginis *virulence factors.

## Background

*Phoma medicaginis *is the causal agent of spring black stem and leaf spot in alfalfa (*Medicago sativa*), a major fodder and forage crop in temperate and Mediterranean regions. Studies in four north-eastern US states have shown foliar necrotrophs, principally *P. medicaginis*, resulted in estimated average yield losses of over 13%. For harvests where significant yield losses occurred, this figure rose to over 19% [1].

*P. medicaginis *also causes disease on the model legume *Medicago truncatula*, or barrel medic, which is used in Australia in ley rotations to enhance soil nitrogen [2]. In susceptible cultivars, reduction in seed and herbage yields, and almost complete defoliation and premature death has been reported [3,4], with infected cultivars exhibiting an average seed weight reduction of 37.3% [5]. Chemical and cultural control of foliar necrotrophs like *P. medicaginis *has proved to be expensive and inefficient [1], and therefore new resistant cultivars or more efficient antifungal control agents are required.

Since the early 1990s, resistance genes (R genes) against various pathogens and pests have been isolated from important crop species such as barley, tomato and rice [reviewed in Hammond-Kosack et al. [6]], although most of the R genes known today were isolated from model plant Arabidopsis. These tend to represent qualitative dominant resistance associated with simple Mendelian genetics. In fungal pathosystems, such genes confer resistance to biotrophs and hemi-biotrophs. Resistance to necrotrophic fungal pathogens, by contrast, is often quantitative. Not surprisingly, the genetic basis of resistance is poorly understood and relatively few resistance genes have been characterised. Those that have been reported involve specific interactions with host selective toxins (HSTs, reviewed in Wolpert et al. [7]) and are predominantly recessive in nature (i.e. susceptibility is dominant) presumably by loss or alteration of gene(s) encoding HST targets. There is evidence to suggest a HST may promote a hypersensitive-like response by acting through a resistance gene leading to cell death [8,9]. By contrast, of the two necrotrophic resistance genes isolated to date, the race-specific *Hm1 *detoxifying gene in maize [10] is dominant and the *Asc-1 *longevity assurance gene homolog in tomato [11,12] is semi-dominant.

The genetic tools to clone and characterize R genes are not available in most legumes, making it difficult to study the molecular basis of resistance to diseases. *Medicago truncatula*, a close relative of alfalfa, is a model system and possesses a small diploid genome that is currently being sequenced, autogamous genetics and various transformation systems. The significance of *M. truncatula *lies in its susceptibility to a range of hemi-biotrophs and necrotrophs and thus is increasingly being used to study resistance to species such as *Aphanomyces euteiches *[13,14], *Ascochyta lentis*, *Botrytis cinerea *and *B. fabae *[15], *Colletotrichum trifolii *[16], *Mycosphaerella pinodes *[17], *Phoma medicaginis *[18], and *Phytophthora medicaginis *[19].

Sources of resistance to *P. medicaginis *have been previously identified among different Medicago spp. [20], and in the genetically diverse South Australian Research and Development Institute (SARDI) *M. truncatula *core collection [21]. Single-seeded SARDI core collection *M. truncatula *accessions were screened for resistance to three virulent Western Australian *P. medicaginis *isolates, and accession SA27063 was shown to be resistant to *P. medicaginis *OMT5, whereas A17 and SA3054 were highly susceptible [18]. In this study we describe the characterisation of the disease response in these accessions macroscopically and cytologically. The genetic basis of resistance to *P. medicaginis *in *M. truncatula *is determined together with genetic map positions for major loci conferring resistance in two different mapping populations.

## Results

### Macroscopic phenotype of resistant and susceptible accessions

**Figure 1:**
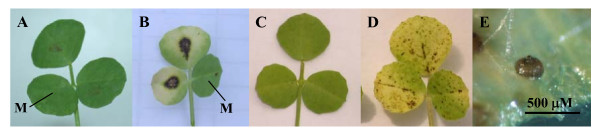
**Macroscopic disease symptoms of *P. medicaginis *OMT5**. **A**, SA27063 spot inoculated 10 days post inoculation (dpi). **B**, SA3054 spot inoculated 10 dpi. **C**, SA27063 spray inoculated 7 dpi. **D**, SA3054 spay inoculated 7 dpi. **E**, Pycnidium of *P. medicaginis *on A17 leaf 14 dpi. **M **indicates mock inoculated leaflets.

*Medicago truncatula *SARDI core collection accessions were previously screened for their response to three *P. medicaginis *isolates. Accession SA27063 was resistant to *P. medicaginis *OMT5, whereas A17 and SA3054 were susceptible [18]. When leaves of three-week-old SA27063 plants were spot inoculated, the fungus was limited to the inoculation site and no chlorosis or necrosis was observed 7–10 days post infection (dpi, Figure 1a). In susceptible interactions the fungus successfully penetrated host cells and spread beyond the inoculation site, accompanied by a halo of chlorosis ahead of the infection zone. By 7–10 dpi infected leaves were entirely chlorotic or necrotic and pycnidia were apparent (Figure 1b, e). Similar symptoms were observed following spray inoculation: small, microscopic hypersensitive response-like lesions and no chlorosis of leaves occurred in resistant SA27063 (10 dpi; Figure 1c); in the susceptible interactions disease symptoms were visible to the naked eye at 7 dpi. *P. medicaginis *colonised the surrounding plant tissue, resulting in macroscopic necrotic spots on both leaves and stems surrounded by spreading chlorosis (10 dpi; Figure 1d). As early as 10 dpi *P. medicaginis *produced pycnidia on susceptible leaves.

### Microscopic disease symptoms of *P. medicaginis OMT5*

**Figure 2:**
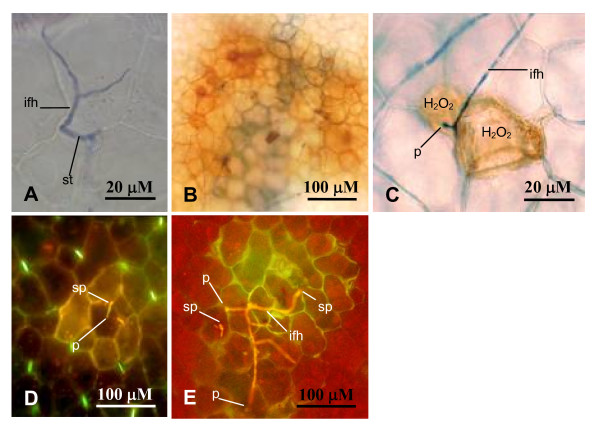
**Microscopic analysis of *P. medicaginis *infection of *M. truncatula***. Leaves were spot inoculated with *P. medicaginis *OMT5 and hyphae visualised with trypan blue (**A-C**) or DiOC6 (**D**, **E**) at 24 and 48 h post infection. (**A**) *P. medicaginis *entering a stomata of SA3054. (**B**, **C**) build-up of H_2_O_2 _around the sites of penetration in SA27063. (**D**, **E**) Autofluorescence of phenolic compounds around the infection site in both SA27063 (**D**) and SA3054 (**E**). H_2_O_2 _= hydrogen peroxide; ifh = infection hyphae; p = point of penetration; sp = spore; st = stomata.

Histological staining using trypan blue or DiOC6 was performed to examine differences in the infection process between resistant and susceptible accessions at the cellular level (Figures 2a–e). Following inoculation, *P. medicaginis *spores germinated on the leaf surface and successful penetration of host cells was observed as early as 6 hours post inoculation (hpi). In both interactions, penetration attempts occurred by three routes; directly through stomata followed by penetration of the underlying mesophyll cells (Figure 2a), directly through the epidermal cells (Figure 2c), or between epidermal cells (Figure 2d). Following penetration, fungal colonisation proceeded rapidly in the susceptible accessions SA3054 and A17, whereas in the resistant accession SA27063 hyphal development was limited to individual or a few epidermal cells. Reddish-brown diaminobenzidine-tetrahydrochloride (DAB) staining to detect endogenous H_2_O_2 _production in cells at or around the site of penetration was observed in accession SA27063 (Figure 2b, c). However, H_2_O_2 _was inconsistently associated with penetration attempts and was also evident in susceptible accessions. Autofluorescence was also observed around the site of infection in both the susceptible and resistant interaction, indicating the production of phenolics (Figure 2d, e).

### Resistance of accession SA27063 to *Phoma medicaginis *OMT5 in two different mapping populations

Accession SA27063 was crossed with susceptible accessions A17 and SA3054 to create two F_2 _mapping populations. F_1 _individuals of both crosses showed *P. medicaginis *OMT5 disease symptoms equivalent to the susceptible parents, indicating recessive resistance. F_2 _individuals were screened for their disease phenotype in both the SA27063 × A17 (n = 92) and SA27063 × SA3054 (n = 94) populations. Both populations differed significantly from a normal distribution (*P*_*W *_< 0.0001; based on the Shapiro and Wilk test [22], which tests the null-hypothesis that a given sample derives from a normally distributed population).

**Figure 3:**
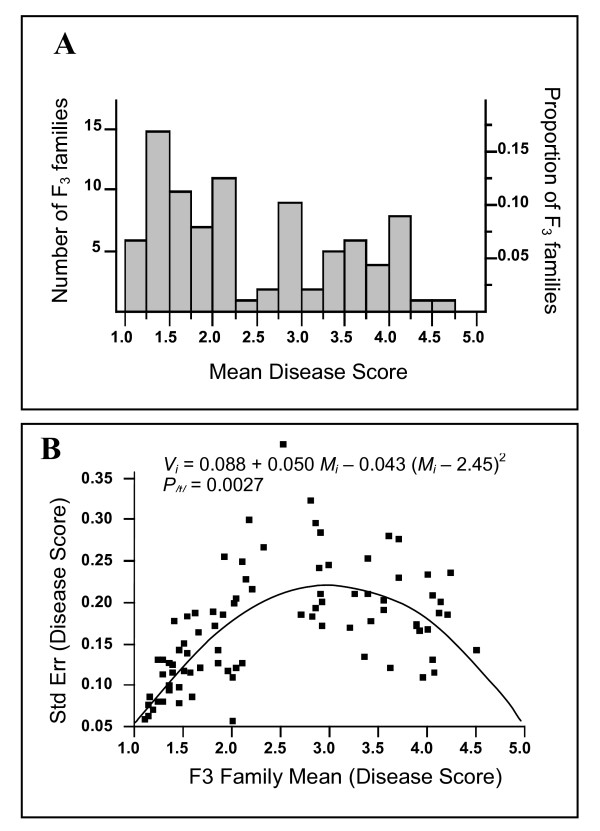
**Response of SA27063 × SA3054 F_3 _families to *P. medicaginis *infection**. **(A) **Frequency distribution of disease scores for F_3 _families of cross SA27063 × SA3054 infected with *P. medicaginis *OMT5 at 7 dpi. (**B) **Variance within a F_3 _family (*V*_*i*_) as a quadratic function of its mean response (*M*_*i*_); (P_/*t*/_) probability testing the null-hypothesis the quadratic term (0.043) is significantly different from zero.

The SA27063 × A17 population was not chosen for further genetic studies due to poor fertility, the presence of a reciprocal translocation between chromosomes four and eight in accession A17 [23], and a large proportion of aberrant F_2 _individuals (Table 1). In the SA27063 × SA3054 cross, F_3 _families (number of individuals per family ≥ 16) were phenotyped to confirm individual F_2 _phenotypes. When the same F_3 _families were inoculated between different experiments, mean disease scores did not alter significantly showing the phenotyping method was reliable and reproducible. The proportions of SA27063 × SA3054 F_3 _families distributed against the mean disease score (Figure 3a) was significantly different from a normal distribution (*P*_*W *_< 0.0001). The relationship between the families' means and the variance within each family is depicted in Figure 3b. This relationship was evaluated using the Fain's test which predicts a pattern of maximum variability in intermediate families whenever at least one locus with major effect is involved [24,25]. Analysis of the F_3 _family disease scores using Fain's test revealed that the quadratic term was highly significant (*P*_/*t*/ _= 0.0027; Figure 3b), indicating one or a few loci with major effects were involved in resistance to *P. medicaginis*.

**Table 1:** Fertility of F_2 _progeny from crosses between A17 and SA27063, and SA3054 and SA27063. Figures are percentages except the average number of seeds per pod, based on 15 randomly selected pods.

	A17 × SA27063 n = 200	3054 × SA27063 n = 200
Pollen viability^1^	47	100
Av. No seeds/pod	3.3	8.3
Albinos	6.2	0
Abnormal radicals	11.8	0
Delayed germination^2 ^	4.9	0
Dwarves	19.3	0

^1^Pollen viability was determined by using Alexanders stain as described in Kamphuis et al. [23].

^2^Delayed germination indicates development at two weeks was equivalent to 48 hrs in a normal seedling.

### Genetic linkage mapping

Genetic maps for the SA27063 × SA3054 and SA27063 × A17 mapping populations were created using both gene-based and microsatellite markers. Markers were initially selected to be evenly distributed over each linkage group and were obtained from several published sources [26-30]. A total of 115 markers were characterised for the SA27063 × SA3054 (n = 94) and 78 for SA27063 × A17 (n = 92) populations.

Forty-six and 58 markers for SA27063 × SA3054 and SA27063 × A17 respectively were single nucleotide polymorphisms (SNPs). The majority of these SNPs were converted to cleaved amplified polymorphic sequences [CAPS, [31]]. SNPs that could not be assayed as CAPS were genotyped using SNapShot (AB) single fluorescent nucleotide extension. A total 53% of markers were polymorphic between SA27063 and A17 and 35% between SA27063 and SA3054. Marker polymorphism data, primer sequences, and references for SA27063 × SA3054 and for SA27063 × A17 markers have been provided [see Additional files 1 and 2].

**Figure 4:**
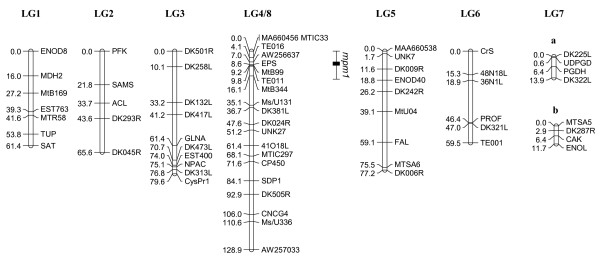
**Genetic map of *M. truncatula *constructed using accessions SA27063 and A17, based on 92 F_2 _individuals**. The genetic map comprises 78 markers and spans 497.8 cM. Horizontal lines show marker positions. Horizontal lines show marker positions and marker names are located to the right. Genetic distances (cM) are located to the left of each marker. The genomic location of the QTL *rnpm1 *for *P. medicaginis *resistance is depicted to the right of LG4, with the standard deviation of its location depicted by lines either side.

**Figure 5:**
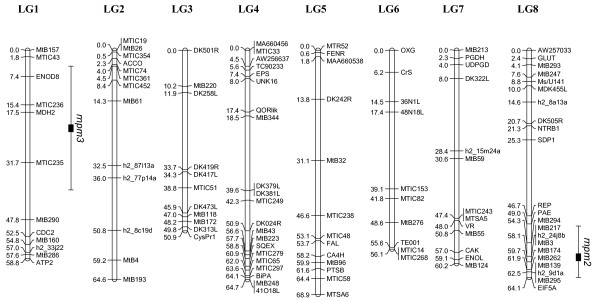
**Genetic map of *M. truncatula *constructed using accessions SA27063 and SA3054, based on 94 F_2 _individuals**. The genetic map comprises 115 markers and spans 488.3 cM. Horizontal lines show marker positions and marker names are located to the right. Genetic distances (cM) are located to the left of each marker. The genomic locations of QTLs *rnpm2 *and *rnpm3 *for *P. medicaginis *resistance are depicted the right of LG8 and LG1 respectively, with standard deviations depicted by lines either side.

The genetic maps for SA27063 × A17 and SA27063 × SA3054 are shown in Figures 4 and 5, respectively. As expected, the SA27063 × SA3054 map was composed of eight linkage groups, in accordance with the basic number of chromosomes in *M. truncatula *(x = 8). The map spans a total of 488.3 centimorgans (cM) with an average distance between markers of 4.2 cM. Very few published markers were polymorphic between SA27063 and SA3054 on the lower half of linkage group 2.

In contrast to the SA27063 × SA3054 map, the SA27063 × A17 map was organized into seven linkage groups, with LG4 and LG8 forming a single linkage group (Figure 4). These linkage groups have previously been reported as single linkage groups [29,30,32]. The establishment of new linkage relationships between LG4 and LG8 combined with observations of semisterility in hybrids involving A17 provides strong evidence that A17 bears a reciprocal translocation, which differentiates it from other *M. truncatula *accessions. This reciprocal translocation involves the lower arm of chromosome 4 and the lower arm of chromosome 8 [23]. All publicly available markers in the middle of LG7 were monomorphic and LG7 was therefore split into two small linkage groups named LG7a and LG7b. The SA27063 × A17 genetic map spans a total of 497.8 cM with an average distance between markers of 6.4 cM. The linkage groups of the genetic maps presented here are similar in length to previously published maps [29,30] with the exception of LG3. In SA27063 × SA3054 the distance between DK501R and CysPR1 was 50.9 cM, whereas in SA27063 × A17 this distance was 79.6 cM.

### QTL mapping of spring black stem and leaf spot resistance in *M. truncatula*

Two major quantitative trait loci (QTLs) for resistance to *P. medicaginis *OMT5 were identified, one in each mapping population. In SA27063 × A17, the effect of the QTL (*d *= 1.4) was significantly different from zero (*P *< 0.00001; LOD = 7.37). The locus was named resistance to the necrotroph *Phoma medicaginis *one (*rnpm1*) and was located on LG4 (Figure 4), tightly linked to AW256637. *rnpm1 *explains 33.6% of the phenotypic variance in the population's response to infection by *P. medicaginis *OMT5, based on a 1–5 scale [18]. In SA27063 × SA3054, the QTL effect (*d *= 1.46) was significantly different from zero (*P *< 0.00001; LOD = 6.77). This locus was named resistance to the necrotroph *Phoma medicaginis *two (*rnpm2*) and was located on LG8 (Figure 5), tightly linked to MtB262. *rnpm2 *explains 29.6% of the phenotypic variance. In SA27063 × SA3054, a second QTL, *rnpm3 *was located on linkage group one. This locus is statistically less significant (*P *= 0.04; LOD = 3.37), has a smaller effect (*d *= 1.1), and explains 19.4% of the total phenotypic variance.

### Fine mapping *rnpm2*

**Figure 6:**
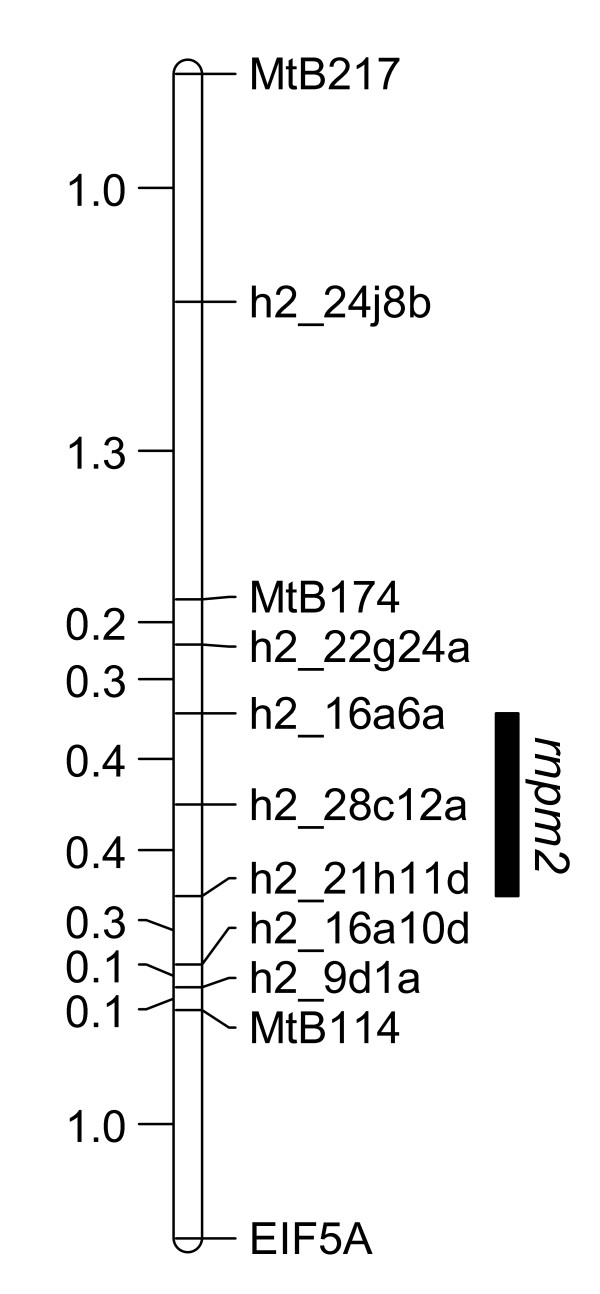
**Fine map genetic position of *rnpm2***. The genomic region encompassing *rnpm2 *for *P. medicaginis *resistance in the SA27063 × SA3054 population is depicted in a 0.8 cM interval between markers h2_16a6a and h2_21h11d on lower LG8. The location is based on a recombination point analysis using recombinant F_3 _families selected from 528 F_2 _individuals.

To initiate fine mapping of *rnpm2 *on the long arm of chromosome eight, an additional 434 F_2 _individuals (524 in total) were screened for recombination within a 8.2 cM interval between markers MtB294, MtB174 and MtB139 (Figure 5). F_3 _families from the selected recombinant F_2 _individuals were phenotypically characterised. As mentioned above, locus *rnpm2 *explains 29.6% of the total variance in the population's response to the fungal infection when measured in a 1–5 scale. However, recombinant families that showed the SA27063-like (resistant) phenotype correlated with homozygous SA27063 alleles at *rnpm2 *in 78.5% of instances. The resistant parent consistently showed little or no visible necrotic symptoms, while disease progression in the susceptible parent was variable and ranged from 2.5 – 4.5. The variance in disease scores was therefore partly a function of the continuum of mean quantitative susceptible disease scores and the sum of disease scores in heterozygous F_3 _families. Recombination break point analysis was therefore used to fine map *rnpm2*, with phenotyping data from thirty-eight informative F_3 _families in conjunction with further polymorphic markers to resolve recombination break points. The location of *rnpm2 *was mapped to a 0.8 cM interval between markers h2_16a6a and h2_21h11d (Figure 6).

## Discussion and Conclusions

The main purpose of this research was to examine differences in disease phenotypes and the genetic basis of resistance to *P. medicaginis *in *M. truncatula*. The resistant phenotype was characterized macroscopically by the presence of small hypersensitive response-like spots, whereas susceptible accessions showed typical stem and leaf lesions, within which pycnidia, or fruiting bodies, formed. In the resistant accession SA27063 pycnidia were not apparent, or where present they developed following a delay of 2–3 weeks, predominantly on senescing leaves. Microscopically, no differences were observed in penetration attempts between resistant and susceptible accessions, and no obvious changes uniquely associated with resistance were evident with the exception of fungal growth being restricted to one or a few plant cells. Production of H_2_O_2 _was observed in resistant SA27063-infected leaves but was also detectable in susceptible SA3054 and A17. H_2_O_2 _has been reported to play an important role in resistance involving a hypersensitive response [33,34]. Build up of reactive oxygen species (ROS) is generally observed following penetration by fungi with a hemibiotrophic or biotrophic lifestyle [16,35] and subsequently leads to resistance against such fungi, while necrotrophic pathogens may stimulate ROS production and subsequent cell death to facilitate subsequent infection [36]. Examination of inoculated leaf tissue also showed accumulation of autofluorescent phenolic compounds around the infection site in both resistant and susceptible interactions. Phenolic compounds, such as flavonoids and isoflavonoids, are an important aspect in legume plant defence [37], and there is ample evidence for their accumulation in response to fungal pathogens in *Medicago *species [38-42]. Pilot transcriptional profiling at 12 hpi using the Mt16kOLI1plus microarray [43] showed significant changes in induced genes related to oxidative burst, cell wall strengthening, lipid metabolism, and the phenylpropanoid pathway leading to isoflavonoid production (L. Kamphuis, unpublished data). However, similar levels of induction occurred in both the resistant and susceptible interactions. Cytological similarities reported here are supported in oats, where cell death and induction of defence-related responses were observed in response to the HST victorin in sensitive genotypes (reviewed by Sweat et al., 2007 [9]), and in barley where a susceptible-specific second ROS burst was observed in the later stages of the infection [44]. Detailed expression and metabolic profiling may help to identify differences in the HR response and the abundance and composition of defence-related compounds and their role in restricting *P. medicaginis *colonisation.

The lower proportion of polymorphic makers in the SA27063 × SA3054 cross suggested this is a narrower cross than SA27063 × A17. The total map length for SA27063 × SA3054 (488.3 cM) was smaller than previously reported genetic maps [29,30,32] and in the SA27063 × A17 map. The genetic distance between markers DK501R and CysPR1 on LG3 in particular was notably smaller than in the SA27063 × A17 map. LG3 corresponds with chromosome three, the largest chromosome of *M. truncatula *[45]. One explanation for this phenomenon could be that accessions SA27063 and SA3054 are sympatric [21], and may share long stretches of homology in chromosome three, resulting in a lack of detectable recombination.

Quantitative trait loci (QTL) analysis is often used to identify and characterise loci conferring resistance. QTL mapping allows the roles of specific *R*-loci to be described, race-specificity of partial resistance genes can be assessed, and interactions between resistance genes, plant development and the environment to be analysed [46]. QTL analysis for *P. medicaginis *resistance in the SA27063 × A17 mapping population revealed a QTL on chromosome four (*rnpm1*) and in the SA27063 × SA3054 population a QTL on chromosome eight (*rnpm2*), both recessive in nature. As explained in the results, although the *rnpm2 *QTL explained a relatively small amount of the total calculated variance for resistance (29.6%), SA27063-like or resistant individuals in the F_2 _mapping population correlated with individuals homozygous for SA27063 alleles at *rnpm2 *in approx. 80% of instances. Among the F_3 _families used in fine mapping and predicted to be resistant by their genotype, this proportion remained constant, allowing *rnpm2 *to be mapped to a 0.8 cM interval between markers h2_16a6a and h2_21h11d by recombination break point analysis. The proportion of resistant homozygous SA27063 *rnpm2 *individuals suggested *rnpm2 *is epistatic to one or several cis- or trans-acting genes or regulatory elements, possibly locus *rnpm3 *on LG1. As the detection and accuracy of minor QTLs in segregating populations largely depends on map quality and population size, increasing the initial mapping population size or genome-wide analysis of expressed QTLs [47] may reveal other minor QTLs and will enable thorough examination of the interaction between *rnpm2 *and *rnpm3*.

We did not proceed with fine mapping *rnpm1 *in the SA27063 × A17 cross, due to poor fertility and a large proportion of aberrant phenotypes among the F_2 _progeny (Table 1), which may cause biased representation of particular gamete genotypes. Furthermore, accession A17 bears a reciprocal translocation, involving chromosomes four and eight, and exhibits reduced pollen viability [23]. All previously published *M. truncatula *genetic maps used accession A17 or a closely related line, J6, as one of the parents. The SA27063 × SA3054 genetic map is the first map produced in *M. truncatula *not involving A17 as a parental line and is therefore useful in identifying the correct position of ambiguously placed markers in A17-derived genetic maps. The reciprocal translocation involves markers distal to *rnpm2 *at approximately 30 cM or less and is therefore unlikely to affect the detection of this QTL among SA27063 × A17 progeny.

The simplest explanation for the existence of two separate QTLs among progeny in two crosses involving the same resistant parent is that genotype-specific susceptibility loci may interact with undetermined *P. medicaginis *OMT5 virulence factors or HSTs. The *rnpm1 *locus is tightly linked to marker AW256637 on BAC AC144658, which is in a contig containing a cluster of TIR-NBS-LRR and disease resistance protein-like genes [48], while no RGAs are apparent in the genomic sequence of the reference accession A17 at the *rnpm2 *locus. The recessive host genotype-specific nature of *rnpm1 *and *rnpm2 *bears similarities to the race-specific *Pyrenophora tritici-repentis *toxin insensitivity QTLs in wheat [49], where multiple, isolate-specific HSTs interact with a given host genotype to produce a susceptible phenotype. To date, no *P. medicaginis *phytotoxins have been characterised *in vivo *during infection. However, the presence of chlorotic symptoms in susceptible *M. truncatula *accessions inoculated with *P. medicaginis *in advance of the fungal hyphae, and the large number of toxins produced by other *Phoma *species suggests that susceptible plants with *Rnpm1 *and *Rnpm2 *are sensitive to a toxin and variation in these genes (*rnpm1 *and *rnpm2*) results in lack of sensitivity.

Future work will be directed at further fine mapping and isolation of *rnpm2*, in conjunction with gene expression studies to identify genes controlling resistance and candidates for *rnpm2 *in the region of interest. In addition, the possible involvement of fungal phytotoxins during infection will be characterised and their interaction with the resistance QTL evaluated.

## Methods

### Growth conditions and mapping populations

To ensure even germination, seeds were scarified with sand using a mortar and pestle, transferred to a Petri dish lined with blotting paper, and irrigated with sterile water. The seeds were kept at 4°C for 48 h, and germinated at room temperature overnight. Seedlings were sown in vermiculite (Richgro Garden Products, Jandakot, Western Australia 6164), fertilized twice a week using Optigrow fertilizer (Growth Technology, O'Connor, Western Australia 6163) and grown under natural light in a temperature controlled greenhouse (22°C day, 18°C night).

Parental *M. truncatula *accessions were identified from the disease phenotype screens described by as being either resistant (SA27063) or susceptible (SA3054 and A17) to *P. medicaginis *OMT5 [18]. Crosses between SA27063 and either A17 or SA3054, as pollen donors, were obtained by a manual crossing procedure [32]. F_1 _seeds were collected and heterozygosity verified with genetic markers polymorphic between the parental accessions.

### Inoculation procedures and resistance evaluation

*P. medicaginis *OMT5 inoculum was prepared by growing the isolate on wheat meal agar plates (WMA, 12 g ground wheat meal, 12 g agar, and 1 litre of distilled water). The plates were incubated at 22°C for 28–42 days under U/V. Conidia were harvested by incubating plates with 10 ml milliQ water for 20 min. and the suspension filtered through a glasswool syringe. Inoculations of were performed at the fourth trifoliate stage. Two methods of inoculation were used: spraying with an artists airbrush to runoff (2 × 10^6 ^spores/ml, 0.05% Tween 20), using a rotating platform to ensure an even distribution over plant surfaces; and spot inoculation with 10 μl droplets of spore suspension (1 × 10^6 ^spores/ml, 0.05% Tween 20) on five leaves per plant. These were the unifoliate, two leaflets of the first trifoliate, and two leaflets of the second trifoliate. The third leaflets of the second and third trifoliates were mock inoculated. To ensure high humidity to stimulate conidia germination, plants were placed in sealed propagator trays for 48 hours.

F_2 _individuals were spot inoculated and macroscopically evaluated at 7 days post inoculation (dpi), then rescored at 10 dpi to confirm more resistant disease reactions as on a 1 (resistant) – 5 (susceptible) scale described by Ellwood et al. [18]. F_3 _families were spray inoculated using a minimum of 16 F_3 _individuals per family and a randomised plot design with two representative F_3 _individuals per family per plot. Disease symptoms were evaluated at 7 and 10 dpi according to the 1–5 scale devised by Salter and Leath [50]. All inoculations incorporated parental control accessions.

### Cytology of the *M. truncatula-P. medicaginis *pathosystem

Three different staining methods were used to study the *M. truncatula *– *P. medicaginis *interaction microscopically:

1) Superficial fungal growth was observed by staining with fluorescent dye 3'3-dihexyloxacarbocyanine iodide (DiOC6_(3)_, Sigma-Aldrich). Whole or part-leaf samples were immersed in fresh aqueous solutions of the fluorescent dye 3 DiOC6_(3) _at 50 mg mL^-1^, prepared from DiOC6_(3) _stock solution in ethanol (0.5 mg mL^-1^, stored at -20°C as described by Duckett and Read [51]. Between 2–3 minutes of exposure normally gave an adequate level of stain absorption. The samples were then placed on slides and the excess stain solution drawn off gently from the edges with tissue. The samples were examined under UV with a light fluorescent Olympus BH-2 microscope fitted with an epifluorescence filter B2A (450–490 nm excitation filter and a 520 nm barrier filter). Hyphae stained bright yellow, conidiospores stained bright green to yellow with increasing age, plant cells exhibiting a hypersensitive response were dark brown or dark green, and healthy cells appeared a deep red colour due to the autofluorescence of intact chloroplasts. Yellow autofluorescence of phenolic compounds could also be observed under UV-light.

2) Superficial and intracellular fungal growth was observed using trypan blue staining. Leaves were fixed in Farmers Fluid until completely cleared. The cleared leaves were immersed in a 0.03% trypan blue stain concentrate mixed with equal volume of 100% ethanol for 30 minutes, then destained in 2.5 g/mL chloral hydrate and examined under a Olympus BH-2 light-microscope.

3) To visualize production of H_2_O_2 _in response to *P. medicaginis *penetration, a DAB (Diaminobenzidine-tetrahydrochloride) staining solution was used [34]. The petioles were placed in the DAB staining solution (1 mg DAB/mL H_2_O pH 3.8, Sigma) for 48 hours. The leaves were fixed and cleared in a solution of ethanol: chloroform (v/v 4:1) and 0.15% TCA for 2–3 hours. After DAB staining leaves were stained with Trypan Blue staining solution for 5–10 minutes. The stained leaves were examined under an Olympus BH-2 light-microscope.

### Statistical analysis of the disease resistance data

Departure from normality for the distribution of disease scores was tested using the *W *test of Shapiro and Wilk [22]. Fain's test was used to evaluate the relationship between the means of the F3 families and the variance within the families [25]. This test assumes that if resistance is determined by one or a few genes with a large effect, that families possessing the most extreme phenotypes are likely to be homozygous, exhibiting low variance within the family. Families with intermediate phenotypes are more likely to be heterozygous, exhibiting large variances (for resistance) within each family. The relationship between families can be described using a quadratic equation, where a significant value (*P*_/*t*/ _< 0.05) indicates the presence of one (or a few) major genes. All the statistical analysis of the data described above was carried out using JMP-IN 5.1 software (SAS Institute, Cary, NC).

### Identification of polymorphic DNA markers

Different types of PCR markers were used to identify polymorphic markers; EST-based intron targeted markers [30]; microsatellites located in introns [28]; microsatellites from sequenced BACs [52] and other published sources [26,29].

Temperature gradient PCR was used to identify the optimum annealing temperature for each primer pair using the standard reference accession *M. truncatula *A17 as a positive control. The following basic PCR protocol was used with minor variations: 50 ng of genomic DNA template, 1 × PCR reaction buffer, 2 mM MgCl_2_, 0.25 mM of each dNTP, 10 pmol of each primer and 1 unit of Taq DNA polymerase. Thermocycling conditions (with minor variations) were: 2 minutes initial denaturation at 94°C followed by 36 cycles of 94°C for 30 sec, marker-specific annealing temperatures for 30 sec, 72°C for 60 sec, then a final extension step of 5 min at 72°C. Where no length or published restriction enzyme polymorphisms were available, PCR products with a single amplicon were direct sequenced using BigDye 3.1 Terminator Cycle Sequencing Ready Reaction Mix (Applied Biosystems [AB], Foster City, California) and the products run on an AB Prism 3730 DNA sequencer. Polymorphisms in the DNA sequences were identified by aligning the sequences in Vector NTI Suite 9.0 (Invitrogen, Carlsbad, California). Where restriction enzymes recognising differences in DNA sequence were available, markers were run as co-dominant CAPS [31]. Single Nucleotide Polymorphisms (SNPs) for which no restriction enzyme was available were detected using SNaPshot (AB), and analysed using GeneMapper v. 3.7 (AB). Large fragment size polymorphisms were resolved by agarose gel electrophoresis and visualised using ethidium bromide staining. Small fragment size polymorphisms were resolved by native polyacrylamide gel electrophoresis or by AB Prism 3730 capillary sequencer using fluorescently labelled primers and a GeneScan™ 500 LIZ^® ^Size Standard (AB).

### Genotyping and linkage analysis

Genomic DNA from the parents and F_2 _individuals were extracted as previously described [21] and polymorphic markers genotyped as described above. A Pearson Chi-square analysis was applied to test the observed segregation ratio of parental alleles against the expected Mendelian segregation ratio for co-dominant inheritance in a F_2 _population, 1:2:1, to remove highly distorted markers. Marker order and map distances were calculated with Multipoint v1.2 software [53] using a maximum recombination fraction (rf) of 0.210 and the Kosambi mapping function. To test the stability of the order of markers for each linkage group (LG), a Jackknife re-sampling approach was used with 5000 iterations. Markers that violated monotonic increase of rfs (i.e. deviation from the expected increase of rf between a marker and its subsequent neighbours) were detected and removed using the control of monotony function. Marker orders were accepted if the probability was greater than 0.90. Removed markers were re-attached to the genetic map in the interval of most probable fit (e.g. minimum increase in the number of recombination events).

### QTL analysis

QTL analysis was performed with MultiQTL v2.5 [53] by applying a general interval mapping and marker restoration method as described by Lichtenzveig et al. [54]. The hypotheses that a single locus or two linked loci have an effect on resistance to *P. medicaginis *were evaluated. Firstly, 5000 permutation tests were performed on the hypothesis that one locus on a chromosome has an effect on the disease resistance (H_1_) versus the null hypothesis (H_0_) that the locus has no effect on the disease resistance. Secondly, 3000 permutation tests were run on the hypothesis that a single locus has an effect on disease resistance versus two linked loci. The model with the highest LOD score was fitted to the QTL and when the models did not differ significantly the simpler model was chosen ('one locus-one trait'). Five thousand bootstrap samples were run to assess the estimates and the standard deviation (SD) of the main parameters: locus effect, its chromosomal position, its LOD score and the proportion of explained variability (PEV).

## Authors' contributions

SE and RPO designed research. LK and SE conducted disease screens, phenotyping, polymorphism discovery, genotyping and genetic mapping. JL provided advice on statistical analyses. LK, JL and SE analysed the data.

## Supplementary Material

Additional file 1 PCR markers used to generate a genetic map in an F_2 _population between accessions SA27063 and SA3054 Click here for file

Additional file 2 PCR markers used to generate a genetic map in an F_2 _population between accessions SA27063 and A17 Click here for file
