# Supplementary material for: Two alternative recessive quantitative trait loci influence resistance to spring black stem and leaf spot in Medicago truncatula
Source: BMC Plant Biol. 2008 Mar 26;8:30. doi: 10.1186/1471-2229-8-30 (PMC2324085; doi:10.1186/1471-2229-8-30)
Supplement: Additional file 1 — PCR markers used to generate a genetic map in an F2 population between accessions SA27063 and SA3054 [file 1471-2229-8-30-S1.doc]

| Marker name | Sequenced amplicon accession number | Linkage Group | Restriction enzyme/fragment size and SNP primer/Fluorescent primers | Method | SA27063 restriction fragment pattern of CAPS or SNP position | SA3054 restriction fragment pattern of CAPS or SNP position | Forward primer sequence | Reverse primer sequence | Reference |
| --- | --- | --- | --- | --- | --- | --- | --- | --- | --- |
| 36N1L |  | 6 | NA | Length | 410 | 950 | GAAGCAGCCGGACATTGGACACA | TGTTAGTTCAAATGGATCTTCTATGAGGTAT | Choi et al 2004 |
| 41O18L |  | 4 | Alu I | CAPS | 268 | 167+101 | AGATATATCAGAAAAAACTAACCCAACCTT | AATACCCTTCCCTTTCCTTCCC | Choi et al 2004 |
| 48N18L |  | 6 | NA | Length | 580 | 480 | TCTTTTCCTCCGATTTCTTGATTCTC | AGGCTTGCTTGCTGTTGGTTGTA | Choi et al 2004 |
| ACCO |  | 2 | Mnl I | CAPS | 40 + 250 | 290 | GAAGATGGCGCAAAAAGAAAGT | CGATGTGTCGTGTCATCTCGTTAAGTTCCCT | Choi et al 2004 |
| ATP2 | DX922473 DX922532 | 1 | Rsa I | CAPS | 950 + 650 +520 | 950 + 520 + 470 + 180 | ATTGCTATGGATGCTACTGAAGGTGTTG | TGGTATGGTGCAAGCAGGTCAA | Choi et al 2004 |
| AW256637 |  | 4 | NA | Length | 600 | 525 | TTCACCTAATTTCCATCTATACCATCCATGT | TATTTGTTAGCTTTAGTGATCGCTGCTACAC | Choi et al 2004 |
| AW257033 | DX922475 DX922533 | 8 | Mae III | CAPS | 314 | 260 + 54 | TGCGTCATTAACCAAAGATGATGTTGTAA | CCAACAGTAACATCCCCAAAGACAATATTC | Choi et al 2004 |
| BiPA | DX922476 DX922534 | 4 | Hinf I | CAPS | 250 +150 | 400 | GAGGAGTCTCACAAAGGATTGC | GGTTTTTCATGTTGTAGACATAGGTTTCA | Choi et al 2004 |
| CA4H | DX922477 DX922535 | 5 | NA | Length | 712 | 483 | GTTACGGGTGGGAGTCTGAA | AGTCCATCATTGCTCGTGGT | This paper |
| CAK |  | 7 | Apo I | CAPS | 545+210+110 | 345 + 200 + 210 + 110 | TTCAACCCCTCTGCGAACC | CATCTATAGCAATTGCTGTTGTCATCT | Choi et al 2004 |
| CDC2 | DX922478 DX922536 | 1 | Dde I | CAPS | 490 +330 | 820 | CAACTTTGCAAGGGTGTTGCTTTCT | ACTAACACCTGGCCACACATCTTCA | Choi et al 2004 |
| CrS |  | 6 | NA | Length | 950 | 750 | CAAATGGTGCTTTGGAGATTGAT | TTAAAAAAGTAGACTGAAGTGTTGACCA | Choi et al 2004 |
| CysPR1 | DX922481 DX922538 | 3 | 197, GTGGCTTCTTATCCCACTAAAAA | SNP | G/59/R | C/59/R | GAGAATTCAAAGAAGAAATTAAGACAAAGA | GAAGAATTCATGGGGAGCAAAGT | Choi et al 2004 |
| DK024R | DX922483 DX922539 | 4 | 440, TTTTGGGGCCCAAAATATAT | SNP | A/313/R | T/313/R | GCCGCGCCATCTTTATTGA | GACGATTTTACCCTTTATCTAAGC | Choi et al 2004 |
| DK242R |  | 5 | Hinc II | CAPS | 330 | 230 + 100 | CGTATGTTTAATCCGTTAGTCCGTCTT | GCTTGCTTAGATATTTGGCACTTCA | Choi et al 2004 |
| DK258L |  | 3 | Xba I | CAPS | 100 + 360 | 470 | GTATTCAGGGATTGAGTAAGAAAAAGGA | ACAAAATCCGTGGATGTATAAAAGTGTA | Choi et al 2004 |
| DK313L |  | 3 | NA | Length | 240 | 265 | GCCAAACATAGGCTAAGTGTGAAAAA | TGACACATAAATTGTTAGCATCTGAAGG | Choi et al 2004 |
| DK322L |  | 7 | Dde I | CAPS | 90 + 30 + 145 | 90 + 175 | GGACCGAACTGGGTCAACAAT | GCACCGAGATCCACCAACAACTT | Choi et al 2004 |
| DK379L | DX922485 DX922540 | 4 | Bsl I | CAPS | 410 | 205 + 205 | AGCTTGTTGAGGTGGAAGGAAGTC | GTGTGTATGAGTGTCGTAAGCCCCT | Choi et al 2004 |
| DK381L | DX922486 DX922541 | 4 | Mae II | CAPS | 330 | 210 + 120 | TGTTACAAAAAGAGTTGGTTGTCGTTC | GTGCACTTTTCAATTTGTCCATCATA | Choi et al 2004 |
| DK417L | DX922487 DX922542 | 3 | 406, CATAAGCTAGAAGGTGCCTTAAC | SNP | T/353/F | A/353/F | ACTCGTCGCCTAACAATATCAACCAG | GAATTCCATATCCAACACCTTTAGACTTA | Choi et al 2004 |
| DK419R | DX922488 DX922543 | 3 | Hae III | CAPS | 420 | 250 + 170 | CATCAGAAGTTGCCAAGCTATCAGAG | GCTTTGGTGCCGTTGTCAGAAGTA | Choi et al 2004 |
| DK455L | DX922489 DX922544 | 8 | 325, GGATGGTAAAGTCCCTGATTGA | SNP | A/97/R | G/97/R | AAGGTTGTGTTGCAGGCGGTTTTAGT | CACATACAGAGTTTCCAGGATTACCATT | Choi et al 2004 |
| DK473L |  | 3 | Bcl I | CAPS | 130 + 230 | 360 | AACTGGTTAACTCGCTAATTGCTACATA | CAATCCTAAACCTCCCAAAAAGC | Choi et al 2004 |
| DK501R |  | 3 | Apo I | CAPS | 420 | 200 + 220 | TATTTGGGATGGAAGCTATGTTGATTGG | TGCTTTAAAGGAGAAGGTAGATGATGAT | Choi et al 2004 |
| DK505R |  | 8 | Ase I | CAPS | 380 | 200 +180 | GCCGCCGCTCCCAAACTT | CAATTCCCTCCGGCGTCACTT | Choi et al 2004 |
| EIF5A | DX922490 DX922545 | 8 | Fluorescent | Length | 1052 | 1038 | CGCGCAGAGAAAGCATCAA | CACAATTGTGGGACGAAGGAAC | Choi et al 2004 |
| ENOD8 |  | 1 | Dra I | CAPS | 876 | 630 + 246 | CCATGCCCATTCCTACTTTTCA | GTGGATTCCACGGACTTTACTTACT | Choi et al 2004 |
| ENOL | DX922492 DX922546 | 7 | Taq I | CAPS | 450 + 1050 | 1500 | TTCCATCAAGGCCCGTCAGA | TTGCACCAACCCCATTCATT | Choi et al 2004 |
| EPS |  | 4 | Bgl II | CAPS | 1050+570 | 1620 | GCTGTTGTGGAAGGCAGTGG | ACGACATACGGAACAGAAATCAGT | Choi et al 2004 |
| EST400 | DX922493 DX922547 | 3 | SfaN I | CAPS | 500 + 500 | 1000 | GGTGGCTGTCCCACTGATTATGT | AAATGCTTGTGTTATGCGGAGAG | Choi et al 2004 |
| FAL |  | 5 | Bcl I | CAPS | 450 | 110 +340 | TTATCGCCAATGCCGCCTACA | ATGATAAGTATGCATGTTCAGAGTCA | Choi et al 2004 |
| FENR | DX922494 DX922548 | 5 | 729, ATTCGGCTACAAGCTATGTAGAA | SNP | T/628/F | C/628/F | ATGCTTATGCCAAAAGATCCAAATGC | CTCACAGCAAAGTCGAGCCTGAAGT | Choi et al 2004 |
| GLUT | DX922497 DX922549 | 8 | 1067, TCGAAGAGAACCACCATCCT | SNP | T/274/F | C/274/F | TACAAGGCAGGGAATCTTAAATCTGCA | TTATTCTCCAGACACCAGCAGTTCCA | Choi et al 2004 |
| h2_15m24a |  | 7 | NA | SSR |  |  | AAAAACACCACATGGCCCTA | GAGGAGGGATCCTTCAAAAA | http://medicago.org/genome/  downloads/Mt_markers_jan06.txt |
| h2_24j8b |  | 8 | NA | SSR |  |  | GCACATGGTTTTGTTGTTGG | GTGAGAGTCACGTTCTGGCA | http://medicago.org/genome/  downloads/Mt_markers_jan06.txt |
| h2_33J22 |  | 1 | NA | SSR |  |  | GATGGATGAGAAATGGAGAA | GAATCAAATCCACCTATCCA | Gutierrez et al 2005 |
| h2_77p14a |  | 2 | NA | SSR |  |  | TGGCGAAATTGACAACACTG | TCACGGTCATCATACTCACTATCC | http://medicago.org/genome/  downloads/Mt_markers_jan06.txt |
| h2_87i13c |  | 2 | NA | SSR |  |  | TGTGGAGATCCTTCATGCAC | TGGCCAAGTGTGTGTTTGAT | http://medicago.org/genome/  downloads/Mt_markers_jan06.txt |
| h2_8a13a |  | 8 | NA | SSR |  |  | GCACTTTTTGGGCTGACTTT | GACAACCGGTCCGAATTTTA | http://medicago.org/genome/  downloads/Mt_markers_jan06.txt |
| h2_8c19d |  | 2 | NA | SSR |  |  | ACAAGGCCTTTAACACCAGC | GGCACTTGTTATCATTTTCCG | http://medicago.org/genome/  downloads/Mt_markers_jan06.txt |
| h2_22g24a |  | 8 | NA | SSR |  |  | cacggtttaggttttcatgg | gctgcattaggttagcagca | http://medicago.org/genome/  downloads/Mt_markers_jan06.txt |
| h2_16a6a |  | 8 | NA | SSR |  |  | ctgccgcatattcagttcat | gtggatcgttggagtgtgtg | http://medicago.org/genome/  downloads/Mt_markers_jan06.txt |
| h2_28c12a |  | 8 | NA | SSR |  |  | ccacgacttgttctccgttt | agcgggtcaggttaggactt | http://medicago.org/genome/  downloads/Mt_markers_jan06.txt |
| h2_21h11d |  | 8 | NA | SSR |  |  | tggttgttagccatccgttt | cctcactgctcaaaaccaca | http://medicago.org/genome/  downloads/Mt_markers_jan06.txt |
| h2_16a10d |  | 8 | NA | SSR |  |  | aaacaaggatgagcggagaa | aggcaaggctcctcgtttat | http://medicago.org/genome/  downloads/Mt_markers_jan06.txt |
| MAA660456 |  | 4 | NA | SSR |  |  | GGGTTTTTGATCCAGATCTT | AAGGTGGTCATACGAGCTCC | Baquerizo-Audiot et al 2001 |
| MAA660538 |  | 5 | NA | SSR |  |  | ATCAAAGCAGAGCAATTTAA | GAAATGCTGTAGGTATCTCC | Baquerizo-Audiot et al 2001 |
| MDH2 |  | 1 | Dra I | CAPS | 70 + 1180 | 70 + 100 + 1080 | CTTCCATTTTCGATTCCTTTCATT | GCATGCCTCGACAACATCAGT | Choi et al 2004 |
| Ms/U141 |  | 8 | Alu I | CAPS | 750 | 530 + 220 | TTGATCAGCCACAGAAATATAAACCA | GCCTCCCACAAAGTAACAAGTTTC | Choi et al 2004 |
| MtB118 |  | 3 | NA | SSR |  |  | GATGCCATCTTTTCTTCAAT | ATCTTTCCGCTAGTGTGTGT | Mun et al 2006 |
| MtB124 |  | 7 | NA | SSR |  |  | CAAGCTTCAATTCCACAAGT | TGGTGAATTACATGCTCAGA | Mun et al 2006 |
| MtB114 |  | 8 | N/A | SSR |  |  | aatcgcgttgaggtaactgg | ctgcttctcttcccatttcg | Mun et al 2006 |
| MtB157 | DX922503 DX922551 | 1 | 223, CACATAATGCCCATCTGAACA | SNP | T/25/R | C/25/R | TGATCGATTACACCGCAAAA | CGACACATAATGCCCATCTG | Mun et al 2006 |
| MtB160 |  | 1 | NA | SSR |  |  | CTGCAATCAACAATTAATGC | AATCACGGGAAATAAGAAAA | Mun et al 2006 |
| MtB172 |  | 3 | NA | SSR |  |  | TTGTTGACGCAAATGACGAT | ATGCATCACACATGCTAGGG | Mun et al 2006 |
| MtB174 |  | 8 | NA | SSR |  |  | TGTGCAGAGGAATCTATAATGA | CCAGCTACTCTTATTGCGTA | Mun et al 2006 |
| MtB193 |  | 2 | Fluorescent | SSR |  |  | CGATTTCCATGATGAGATTT | ACCATCACACAACATAATTCAC | Mun et al 2006 |
| MtB213 |  | 7 | NA | SSR |  |  | GTCGATTTTGACTCTCTTGC | TGGCCAACTTAGACAAATCT | Mun et al 2006 |
| MtB217 |  | 8 | NA | SSR |  |  | TCGACAGTAATACACGCTCA | GTCTGAAAATCATCCAAAGC | Mun et al 2006 |
| MtB220 |  | 3 | NA | SSR |  |  | AAGGTGTCTTCTAGCAAAACTAA | TCATAACCCGGATCGTAGTA | Mun et al 2006 |
| MtB223 |  | 4 | NA | SSR |  |  | AACGTGAGCTTGTAGCAGCA | ACACCCTTTACCACCACTGC | Mun et al 2006 |
| MtB247 |  | 8 | NA | SSR |  |  | GTGGAAGTTTGATCATTTCG | CAAATCTCACACCCACTACA | Mun et al 2006 |
| MtB248 |  | 4 | NA | SSR |  |  | CTGTATCGCCAAATGAATTA | TAAACAGAGCCGAACTATCC | Mun et al 2006 |
| MtB26 | DX922504 DX922552 | 2 | 272, GCTAGGATCGCACTCCCTAA | SNP | T/57/R | C/57/R | CGGTTTTGGTGGAGAAGTTG | TCTTAATACCCGTGGGAGCA | Mun et al 2006 |
| MtB262 |  | 8 | NA | SSR |  |  | AAACGAAGCAAGCAGCTCAT | GAAGCAAGAAGAAAAGGAGTGG | Mun et al 2006 |
| MtB27 | DX922505 DX922553 | 4 | Hae III | CAPS | 100 + 100 | 200 | TCCCAACGCTTTTTCATTTC | GAACTTGAAGAAGAACGCCG | Mun et al 2006 |
| MtB276 | DX922506 DX922554 | 6 | Nde I | CAPS | 110 + 140 | 250 | CCCTCCCTTTCCTTTCAAAC | GATGTGCCCCTTTTTCTCAT | Mun et al 2006 |
| MtB286 |  | 1 | NA | SSR |  |  | CTCTTCTCACGCTGATGCTG | TGGTGGAAGGAACGGTTAAG | Mun et al 2006 |
| MtB290 |  | 1 | Fluorescent | SSR |  |  | TTCAAAACAGTGGGGAAAGC | TTGGTTTTGTTCTGCGTCTG | Mun et al 2006 |
| MtB293 | DX922507 DX922555 | 8 | Apo I | CAPS | 220 | 180 + 40 | CAAGCTTCAATTCCACAAGT | TGGTGAATTACATGCTCAGA | Mun et al 2006 |
| MtB294 |  | 8 | NA | SSR |  |  | AATTTCGGACGTTCATTTCG | CCGATTTACAATTTAGACTGCCA | Mun et al 2006 |
| MtB295 |  | 8 | NA | SSR |  |  | AACCTTACGGTGTCGTTTCG | TTTCTCAAACCCATCGATCC | Mun et al 2006 |
| MtB3 |  | 8 | NA | SSR |  |  | CACATAACAACCACCACCACA | CCCAGGTTGTTGAGGAAGAA | Mun et al 2006 |
| MtB300 |  | 1 | NA | SSR |  |  | ATCTGGTAGGAGATGGTGCG | ATGCAGAGGGGTGATTCAAG | Mun et al 2006 |
| MtB32 | DX922508 DX922556 | 5 | BsmA I | CAPS | 610 | 490 + 120 | GGATTCAGGACCAAGAGACT | CAAAATGCCACTGTTATAAGG | Mun et al 2006 |
| MtB344 | DX922509 DX922557 | 4 | 198, CCACCGCTCAAGTTAGTATTTG | SNP | A/101/F | C/101/F | ATGGAGAAGATGTTCCGACG | GTCACACCAGGTGCACAATC | Mun et al 2006 |
| MtB4 |  | 2 | NA | SSR | 420 |  | GCCCTAAGGACTGCATTTTG | CCCCTCCTAAACCCTCAATC | Mun et al 2006 |
| MtB43 |  | 4 | NA | SSR |  |  | GCCCTAAGGACTGCATTTTG | CCCCTCCTAAACCCTCAATC | Mun et al 2006 |
| MtB55 |  | 7 | Fluorescent | SSR |  |  | TCTGAAAGGGCACCTCCTAA | CGCGAAAGGAATGTTGAAGT | Mun et al 2006 |
| MtB59 | DX922510 DX922558 | 7 | Mse I | CAPS | 180 +40 | 220 | CCCTTGACTGAAAGGGACAA | TCGGTGTCGAACACCTACAC | Mun et al 2006 |
| MtB61 |  | 2 | Fluorescent | SSR |  |  | CGGACAAAACAGATTGTCCTT | GAAGGTGCGTTTTAGCAACA | Mun et al 2006 |
| MtB96 |  | 5 | NA | SSR |  |  | TAGCTCAATTGGCATGGACA | TGGCAAACGTTACCAAACAA | Mun et al 2006 |
| MTIC014 |  | 6 | NA | SSR |  |  | CAAACAAACAACACAAACATGG | CCCATTGATTGGTCAAGGTT | Gutierrez et al 2005 |
| MTIC019 |  | 2 | NA | SSR |  |  | TCTAGAAAAAGCAATGATGTGAGA | TGCAACAGAAGAAGCAAAACA | Gutierrez et al 2005 |
| MTIC033 |  | 4 | NA | SSR |  |  | AAAATTAGAAGAACCACGGCTTT | AATCGCTTTCCCAATTTCAA | Gutierrez et al 2005 |
| MTIC051 |  | 3 | NA | SSR |  |  | AGTATAGTGATGAAGTGGTAGTGAACA | ACAAAAACTCTCCCGGCTTT | Gutierrez et al 2005 |
| MTIC065 |  | 4 | NA | SSR |  |  | ATGCTTGCAAGGGGTCTCTA | AATGCATACACCAAACTTAAACATT | Gutierrez et al 2005 |
| MTIC153 |  | 6 | NA | SSR |  |  | TCACAACTATGCAACAAAAGTGG | TGGGTCGGTGAATTTTCTGT | Gutierrez et al 2005 |
| MTIC235 |  | 1 | NA | SSR |  |  | CCTTTGGTTGATTCAGTTTC | CCAATATGTCACTCCTTGCT | Gutierrez et al 2005 |
| MTIC236 |  | 1 | NA | SSR |  |  | AAGTCATCAGGACCAACAAC | TGTTTGACGCAAAGAAAGAT | Gutierrez et al 2005 |
| MTIC238 |  | 5 | NA | SSR |  |  | TTCTTCTTCTAGGAATTTGGAG | CCTTAGCCAAGCAAGTAAAA | Gutierrez et al 2005 |
| MTIC243 |  | 7 | NA | SSR |  |  | GGAGGAGGTTATAGGTTTGG | TCAGTGCTCAGCATCTATGT | Gutierrez et al 2005 |
| MTIC249 |  | 4 | NA | SSR |  |  | TAGGTCATGGCTATTGCTTC | GTGGGTGAGGATGTGTGTAT | Gutierrez et al 2005 |
| MTIC268 |  | 6 | NA | SSR |  |  | GAGGATTCATTCTTCTTCCA | ATTGTTCCTAGGTTGGGTTT | Gutierrez et al 2005 |
| MTIC279 |  | 4 | NA | SSR |  |  | GCAGCACAAGATACTCACAA | CTTAGACGGTGTTGGTTTTC | Gutierrez et al 2005 |
| MTIC297 |  | 4 | NA | SSR |  |  | CTAAGCTTTGGCCATGTATC | TGAAATGAGTTTGACTGAGG | Gutierrez et al 2005 |
| MTIC354 |  | 2 | NA | SSR |  |  | AAGTGCCAAAGAACAGGGTTT | AACCTACGCTAGGGTTGCAG | Gutierrez et al 2005 |
| MTIC361 |  | 2 | NA | SSR |  |  | AGCTGAAGTGGAACCACCAG | CCCCTAGCTTGAGGAGAGGA | Gutierrez et al 2005 |
| MTIC43 |  | 1 | NA | SSR |  |  | CGTCGTTACTCAAACGACACC | GCGTGTTTCTGCTGATTCAT | Gutierrez et al 2005 |
| MTIC452 |  | 2 | NA | SSR |  |  | CTAGTGCCAACACAAAAACA | TCACAAAAACTGCATAAAGC | Gutierrez et al 2005 |
| MTIC48 |  | 5 | NA | SSR |  |  | TTTTTGTTAGTTTGATTTTAGGTG | GCTACAAAGTCTTCTTCCACA | Gutierrez et al 2005 |
| MTIC58 |  | 5 | NA | SSR |  |  | AGGTGGGCACAACAAAAGAG | CTCTCCATTTTCACCGCTTC | Gutierrez et al 2005 |
| MTIC74 |  | 2 | NA | SSR |  |  | GGTGGAAGGAACAACTCTGG | CCGGCATGATTAAGACACAC | Gutierrez et al 2005 |
| MTIC82 |  | 6 | NA | SSR |  |  | CACTTTCCACACTCAAACCA | GAGAGGATTTCGGTGATGT | Gutierrez et al 2005 |
| MTR52 |  | 5 | Fluorescent | SSR |  |  | AGCGGATGGTGGGGATA | ATTCCATTTACTGCTTATCG | Baquerizo-Audiot et al 2001 |
| MTSA5 |  | 7 | NA | SSR |  |  | ACTGTTCCGTCCTTTCAATC | TGAGTTCTTGTTCCTTGTTA | Baquerizo-Audiot et al 2001 |
| MTSA6 |  | 5 | NA | SSR |  |  | TCACATTAATTATCTTTTCACAA | GGCCAAAACATAAAAATTG | Baquerizo-Audiot et al 2001 |
| NTRB1 | DX922511 DX922559 | 8 | Dde I | CAPS | 560 | 470 + 90 | CACGACTCTGCACGCTTTGTTA | ACCCTTGTTGCGAGTCATTTG | Choi et al 2004 |
| OXG | DX922512 DX922560 | 6 | Rsa I | CAPS | 720 | 390 + 330 | AGGTGTAGCAAGATATAACCAATTCAGGA | TTTGGTGGTGCATCCCAAACAGAGAAAG | Choi et al 2004 |
| PAE | DX922513 DX922561 | 8 | 800, CTTTTGTCTTGTAATGATCTTGAGT | SNP | A/742/F | T/742/F | CTAAAAGCAGCAGAAGGGGTTAC | GATCCGGTCAAGGCAAGTAGTT | Choi et al 2004 |
| PGDH |  | 7 | Bcl I | CAPS | 460 + 30 | 490 | GAGTTGAAGCTGCAAAGGTCTTTAAATCA | TGTATGAGCACCGAAGTAGTCTCGTTGA | Choi et al 2004 |
| PTSB | DX922516 DX922562 | 5 | Fluorescent | Length | 365 | 370 | ACTAAACAACACGCTAATTGGTCTCCA | ATGCCTAGCAGACAAAACCTTCTGCA | Choi et al 2004 |
| QORlik | DX922517 DX922563 | 4 | 409, TGTGTATGTATCTCCAAGTCTACT | SNP | C/261/F | A/261/F | GATGGTCTGGCAACTGT | AGGGAGGACTTTTCTTAG | Choi et al 2004 |
| REP | DX922518 DX922564 | 8 | Mnl I | CAPS | 300 + 50 + 65 | 350 + 65 | CTCCATTTCCCGTTCGTTCG | CACCGGTTGCCCTCCAGAC | Choi et al 2004 |
| SDP1 |  | 8 | Ssp I | CAPS | 76+829+62 | 76+726+103 + 62 | TGGCTCTAAATCAGGGGAAGAATA | TGTGACGGTTGAATATCTGAATGTTT | Choi et al 2004 |
| SQEX | DX922521 DX922565 | 4 | Xba I | CAPS | 880 + 190 | 1070 | TGCCGCTATAAAAAGTAAACAAAGAA | CAATTCACCCACAATTCTATCAGG | Choi et al 2004 |
| TC90233 | DX922522 DX922566 | 4 | Alu I | CAPS | 100 + 120 | 220 | CCGATCCAAAGAAGAGAAGG | GAACACAGGCAGAACAACCA | This paper |
| TE001 | DX922523 DX922567 | 6 | Bcc I | CAPS | 520 + 380 | 380 + 440 + 80 | CGGCGCCGGAGATTACACTG | AATCACAAACCCACCCAACATCTG | Choi et al 2004 |
| TUP |  | 1 | Bcl I | CAPS | 620 | 230 + 390 | GAATGGGATGCTATGGGAAGTG | TGGATCAGTGGCACCATCTTTAT | Choi et al 2004 |
| UDPGD | DX922527 DX922568 | 7 | Mbo II | CAPS | 825 + 400 + 105 | 650 + 400 + 175 + 105 | CAAAAGCGTTTCATCACTCATCTCT | ATCGTCAAGGCCAGGTTCATAG | Choi et al 2004 |
| UNK16 | DX922528 DX922569 | 4 | 322, AAATCCAGGCATAACCATCAA | SNP | C/293/F | T/293/F | CCTTCCAATATCCCTCCCACAT | GAAGAAAATGATGAAAAGCCAAAAG | Choi et al 2004 |
| UNK3 | DX922530 DX922570 | 4 | Alu I | CAPS | 250 + 120 | 370 | CACCGGAAATTCAACAGCAAC | GACCTAGGCAACACAACTCCATTA | Choi et al 2004 |
| VR | DX922531 DX922571 | 7 | Xho I | CAPS | 800 | 600+ 300 | AAGCTGTTCTTGAATTTGGTGA | TCCACGAGTTTCTTCGTGTTT | This paper |
