# Supplementary material for: Two alternative recessive quantitative trait loci influence resistance to spring black stem and leaf spot in Medicago truncatula
Source: BMC Plant Biol. 2008 Mar 26;8:30. doi: 10.1186/1471-2229-8-30 (PMC2324085; doi:10.1186/1471-2229-8-30)
Supplement: Additional file 2 — PCR markers used to generate a genetic map in an F2 population between accessions SA27063 and A17 [file 1471-2229-8-30-S2.doc]

| Marker name | Sequenced amplicon accession number | Linkage Group | Restriction enzyme | Method | SA27063 restriction fragment pattern of CAPS | A17 restriction fragment pattern of CAPS | Forward primer sequence | Reverse primer sequence | Reference |
| --- | --- | --- | --- | --- | --- | --- | --- | --- | --- |
| 36N1L | DX922470 DX922442 | 6 | Hae III | CAPS | 410 | 190 + 220 | GAAGCAGCCGGACATTGGACACA | TGTTAGTTCAAATGGATCTTCTATGAGGTAT | Choi et al 2004 |
| 41O18L | DX922471 DX922443 | 4 | Mse I | CAPS | 210 + 72 | 76 + 134 + 72 | AGATATATCAGAAAAAACTAACCCAACCTT | AATACCCTTCCCTTTCCTTCCC | Choi et al 2004 |
| 48N18L | DX922472 DX922444 | 6 | Apo I | CAPS | 450 + 140 | 590 | TCTTTTCCTCCGATTTCTTGATTCTC | AGGCTTGCTTGCTGTTGGTTGTA | Choi et al 2004 |
| ACL |  | 2 | Bcl I | CAPS | 1200 | 30 + 270 + 900 | AAGGTTAAGACCGTATTTATTCCAACA | AGTCCAAATTCGTCCCCACTG | Choi et al 2004 |
| AW256637 | DX922474 DX922445 | 4 | NA | Length | 574 | 524 | TTCACCTAATTTCCATCTATACCATCCATGT | TATTTGTTAGCTTTAGTGATCGCTGCTACAC | Choi et al 2004 |
| AW257033 | DX922475 DX922446 | 8 | Nla III | CAPS | 280 + 40 + 50 +26 | 320 + 50 + 26 | TGCGTCATTAACCAAAGATGATGTTGTAA | CCAACAGTAACATCCCCAAAGACAATATTC | Choi et al 2004 |
| CAK |  | 7 | Apo I | CAPS | 545 + 210 + 110 | 345 + 200 + 210 + 110 | TTCAACCCCTCTGCGAACC | CATCTATAGCAATTGCTGTTGTCATCT | Choi et al 2004 |
| CNCG4 |  | 8 | Bsl I | CAPS | 380 |  | AGAGATGAGAATCAAGAGGAGGGATGCA | CATGATGAAGAGCATTTCGTCCACTGGA | Choi et al 2004 |
| CP450 | DX922479 DX922447 | 4 | Hae III | CAPS | 260 + 130 + 75 | 390 + 75 | AGTGTGAGATCAATGGTTATGTGATC | CATCATCACCTTTCAATATTTGTCC | Choi et al 2004 |
| CPOX2 | DX922480 DX922448 | 8 | Xmn I | CAPS | 220 + 80 | 300 | GATAATGGCCTTGTTATGAATTACTACA | GCTCAGACAAGCTTCTTCTTGTGGA | Choi et al 2004 |
| CrS |  | 6 | NA | Length | 950 | 750 | CAAATGGTGCTTTGGAGATTGAT | TTAAAAAAGTAGACTGAAGTGTTGACCA | Choi et al 2004 |
| CysPr1 |  | 3 | Acl I | CAPS | 60 + 170 | 230 | GAGAATTCAAAGAAGAAATTAAGACAAAGA | GAAGAATTCATGGGGAGCAAAGT | Choi et al 2004 |
| DK006R | DX922482 DX922449 | 5 | Rsa I | CAPS | 450 | 405 + 45 | GAACATAACCCCGAAGTGGAT | GAGTTTGGGAACAAAATTAGTATGAT | Choi et al 2004 |
| DK009R |  | 5 | Dra I | CAPS | 280 + 170 | 450 | TAGCATCATCTTTCCCATACAA | GGGCAGGCAGCACCAGATA | Choi et al 2004 |
| Dk024R | DX922483 DX922450 | 4 | Bsr I | CAPS | 440 | 315 + 135 | GCCGCGCCATCTTTATTGA | GACGATTTTACCCTTTATCTAAGC | Choi et al 2004 |
| DK045R | DX922484 DX922451 | 2 | Mlu I | CAPS | 390 | 240 + 150 | TGGCAATATCCACCAAATCAAA | CGAACCCACGACCACAAGG | Choi et al 2004 |
| DK132L |  | 3 | Xmn I | CAPS | 290 + 30 | 320 | TGGACCTAAGACTTCAAAGATTCAGA | CCTATTAAGCATATTTGCAGCATGAACAATTT | Choi et al 2004 |
| DK225L |  | 7 | Nco I | CAPS | 60 + 210 | 270 | TGTCCTTGCTTCTTATCCTTCCTTCA | AGCAGCACAACAACTTACAACAACTC | Choi et al 2004 |
| DK242R |  | 5 | Hinc II | CAPS | 330 | 230 + 100 | CGTATGTTTAATCCGTTAGTCCGTCTT | GCTTGCTTAGATATTTGGCACTTCA | Choi et al 2004 |
| DK258L |  | 3 | Xba I | CAPS | 100 + 360 | 470 | GTATTCAGGGATTGAGTAAGAAAAAGGA | ACAAAATCCGTGGATGTATAAAAGTGTA | Choi et al 2004 |
| DK287R |  | 7 | Dde I | CAPS | 135 + 135 | 270 | AGCCGCCCTCTTGAACCTCC | TAGCTGCAACAAAGAAACCAAAACC | Choi et al 2004 |
| DK293R |  | 2 | Dra I | CAPS | 290 | 80 + 210 | ACTTACAAGGTTAGCGTCATTCTCCATC | GCTATCCCACCTTAAAATTTCTTCACAA | Choi et al 2004 |
| DK313L |  | 3 | NA | Length | 240 | 265 | GCCAAACATAGGCTAAGTGTGAAAAA | TGACACATAAATTGTTAGCATCTGAAGG | Choi et al 2004 |
| DK321L |  | 6 | Msl I | CAPS | 370 | 120 + 250 | GAGCGAGCTCAGGATAGACTTTAGAA | TCCCACCTCCAATTTGTAGACGAT | Choi et al 2004 |
| DK322L |  | 7 | Dde I | CAPS | 90 + 30 + 145 | 90 + 175 | GGACCGAACTGGGTCAACAAT | GCACCGAGATCCACCAACAACTT | Choi et al 2004 |
| DK381L |  | 4 | Hinf I | CAPS | 40 + 210 + 80 | 250 + 80 | TGTTACAAAAAGAGTTGGTTGTCGTTC | GTGCACTTTTCAATTTGTCCATCATA | Choi et al 2004 |
| DK417L |  | 3 | Bbv I | CAPS | 180 + 230 | 410 | ACTCGTCGCCTAACAATATCAACCAG | GAATTCCATATCCAACACCTTTAGACTTA | Choi et al 2004 |
| DK473L |  | 3 | Bcl I | CAPS | 360 | 130 + 230 | AACTGGTTAACTCGCTAATTGCTACATA | CAATCCTAAACCTCCCAAAAAGC | Choi et al 2004 |
| DK501R |  | 3 | Apo I | CAPS | 420 | 200 + 220 | TATTTGGGATGGAAGCTATGTTGATTGG | TGCTTTAAAGGAGAAGGTAGATGATGAT | Choi et al 2004 |
| DK505R |  | 8 | Ase I | CAPS | 380 | 200 + 180 | GCCGCCGCTCCCAAACTT | CAATTCCCTCCGGCGTCACTT | Choi et al 2004 |
| DNABP |  | 4 | Afl II | CAPS | 860 + 260 | 280 + 580 + 260 | CCCTATGAGCTTGGGTTTGTCT | CTCATGGCATACGTGTTCAGC | Choi et al 2004 |
| EIF5A |  | 8 | Dde I | CAPS | 360 + 620 | 980 | CGCGCAGAGAAAGCATCAA | CACAATTGTGGGACGAAGGAAC | Choi et al 2004 |
| ENOD40 |  | 5 | NA | Length | 177 | 136 | AACCAATGCCACTTTTCACTTTGCCTCC | AGACTCTTGCGAGTGCTACCATTTGACC | Choi et al 2004 |
| ENOD8 | DX922491 DX922452 | 1 | Pvu I | CAPS | 650 + 250 | 900 | CCATGCCCATTCCTACTTTTCA | GTGGATTCCACGGACTTTACTTACT | Choi et al 2004 |
| ENOL | DX922492 DX922453 | 7 | Taq I | CAPS | 450 + 1050 | 1500 | TTCCATCAAGGCCCGTCAGA | TTGCACCAACCCCATTCATT | Choi et al 2004 |
| EPS |  | 4 | Bgl II | CAPS | 1050 + 570 | 1620 | GCTGTTGTGGAAGGCAGTGG | ACGACATACGGAACAGAAATCAGT | Choi et al 2004 |
| EST400 |  | 3 | Sac I | CAPS | 1000 | 500 + 500 | GGTGGCTGTCCCACTGATTATGT | AAATGCTTGTGTTATGCGGAGAG | Choi et al 2004 |
| EST763 |  | 1 | Hinf I | CAPS | 155 | 130 + 25 | CACTCTAAAAAGGCCCAGAAGGTTTGACT | CTTATGACCAATAGTCTGTTCCACTC | Choi et al 2004 |
| FAL |  | 5 | Bcl I | CAPS | 110 + 340 | 450 | TTATCGCCAATGCCGCCTACA | ATGATAAGTATGCATGTTCAGAGTCA | Choi et al 2004 |
| FIS1 | DX922495 DX922454 | 8 | Apo I | CAPS | 616 + 122 + 90 + 124 + 43 | 488 + 128 + 122 + 90 + 124 + 43 | TCAGTGATTGAGGGTTTTTCTACG | CTGTTTCATCAACTTCAGCAACTTT | Choi et al 2004 |
| GLNA | DX922496 DX922455 | 3 | HindIII | CAPS | 540 | 195 + 345 | GAATGGTGCTGGTGCTCACACA | TGGTGGTGTCTGCAATCATGGAAG | Choi et al 2004 |
| HYPTE3 | DX922498 DX922456 | 4 | BsmA I | CAPS | 180 + 170 | 350 | TCGTCTCATGGTGGAATCGTGATGGT | TTCCTCCTTTAAACAAGCAAATTGGA | Choi et al 2004 |
| MAA660456 |  | 4 | NA | SSR |  |  | GGGTTTTTGATCCAGATCTT | AAGGTGGTCATACGAGCTCC | Baquerizo-Audiot et al 2001 |
| MAA660538 |  | 5 | NA | SSR |  |  | ATCAAAGCAGAGCAATTTAA | GAAATGCTGTAGGTATCTCC | Baquerizo-Audiot et al 2001 |
| MAAP | DX922499 DX922457 | 8 | Afl III | CAPS | 990 | 605 + 385 | TACCTAAGACTGCACATGCTATGTAT | CATCACCAACACGCTTTACAGTGCGGCT | Choi et al 2004 |
| MDH2 |  | 1 | Dra I | CAPS | 70 + 1180 | 70 + 100 + 1080 | CTTCCATTTTCGATTCCTTTCATT | GCATGCCTCGACAACATCAGT | Choi et al 2004 |
| MPP |  | 4 | BstB I | CAPS | 440 | 110 + 330 | TCCCCGAAACAATCCTCATCTG | GCAAATGTGTAGCCCCAAAAGTTA | Choi et al 2004 |
| Ms/U131 | DX922500 DX922458 | 4 | Hinf I | CAPS | 410 | 180 + 230 | ATGCTATTGGGACTCAACACTCTGA | GGAATTGCACTATACAGATGATAGGA | Choi et al 2004 |
| Ms/U336 | DX922502 DX922459 | 8 | Rsa I | CAPS | 560 | 280 + 280 | AGACGTGGCTAACTTCGAAACACT | GAGCTTGAAACATTAGCATTGTTGTTA | Choi et al 2004 |
| MtB169 |  | 1 | NA | SSR |  |  | AGGCTGAAAATGGCTTGAAA | CCACACAGATGCCACAGACT | Mun et al 2006 |
| MtB99 |  | 4 | NA | SSR |  |  | CTTGGCAAAATGTCAACTCT | GGAAAGGGGTTAGGTGAGTA | Mun et al 2006 |
| MTIC033 |  | 4 | NA | SSR |  |  | AAAATTAGAAGAACCACGGCTTT | AATCGCTTTCCCAATTTCAA | Gutierrez et al 2005 |
| MTR58 |  | 1 | NA | SSR |  |  | GAAGTGGAAATGGGAAACC | GAGTGAGTGAGTGTAAGAGTGC | Baquerizo-Audiot et al 2001 |
| MTSA5 |  | 7 | NA | SSR |  |  | ACTGTTCCGTCCTTTCAATC | TGAGTTCTTGTTCCTTGTTA | Baquerizo-Audiot et al 2001 |
| MTSA6 |  | 5 | NA | SSR |  |  | TCACATTAATTATCTTTTCACAA | GGCCAAAACATAAAAATTG | Baquerizo-Audiot et al 2001 |
| MtU04 |  | 5 | Nsi I | CAPS | 1165 | 310 + 855 | ATGGGAAGAGGATTGCTGTGATA | AAGCGAACATTTTTGGCATCTAC | Choi et al 2004 |
| NPAC |  | 3 | Ssp I | CAPS | 470 + 270 + 500 | 340 + 130 + 270 + 500 | TGGCTCCAGGTCCAGTTATTGA | TCGGCTCTTCTTCTCGCTTCT | Choi et al 2004 |
| PAE | DX922513 DX922460 | 8 | Ava II | CAPS | 470 + 330 | 800 | CTAAAAGCAGCAGAAGGGGTTAC | GATCCGGTCAAGGCAAGTAGTT | Choi et al 2004 |
| PFK |  | 2 | Ssp I | CAPS | 80 + 420 | 500 | TCCCACTGCAAATCATGTCAAAAC | ACACAAGTGGATATTGATGGTTAGACTAC | Choi et al 2004 |
| PGDH | DX922514 DX922461 | 7 | Bcl I | CAPS | 460 + 30 | 490 | GAGTTGAAGCTGCAAAGGTCTTTAAATCA | TGTATGAGCACCGAAGTAGTCTCGTTGA | Choi et al 2004 |
| PROF | DX922515 DX922462 | 6 | BsaJ I | CAPS | 195 + 125 | 320 | AGAAGTCAAAAATGGTCTACCAGTGA | CAAATCTTCCAATATCCAAACAAGTAGGA | Choi et al 2004 |
| QORlik |  | 4 | Rsa I | CAPS | 409 | 119 + 209 | GATGGTCTGGCAACTGT | AGGGAGGACTTTTCTTAG | Choi et al 2004 |
| SAMS |  | 2 | BstB I | CAPS | 525 + 525 | 1050 | CATAGCAAAGCGGGTTCAATCT | GTCAGCATCAAGACCAACATCATC | Choi et al 2004 |
| SAT | DX922519 DX922463 | 1 | Dde I | CAPS | 565 + 60 + 195 | 625 + 195 | GTATCATGATGGACTTGATCATTTTCGTC | AGCCTTTGCATGCCACTGCACCTCA | Choi et al 2004 |
| SDP1 | DX922520 DX922464 | 8 | Bcl I | CAPS | 660 + 350 | 1100 | TGGCTCTAAATCAGGGGAAGAATA | TGTGACGGTTGAATATCTGAATGTTT | Choi et al 2004 |
| SQEX |  | 8 | EcoRV | CAPS | 190 + 620 +260 | 810 + 260 | TGCCGCTATAAAAAGTAAACAAAGAA | CAATTCACCCACAATTCTATCAGG | Choi et al 2004 |
| TE001 | DX922523 DX922465 | 6 | Ava II | CAPS | 750 + 250 | 1000 | CGGCGCCGGAGATTACACTG | AATCACAAACCCACCCAACATCTG | Choi et al 2004 |
| TE011 | DX922524 DX922466 | 4 | Hinc II | CAPS | 1550 | 1200 + 350 | GGAGAGAAACCGGACTGAAGAAACA | CAAGAAGAAGCCCTAGTCCTCCATT | Choi et al 2004 |
| TE016 | DX922525 DX922467 | 4 | Hinc II | CAPS | 1950 | 1530 + 420 | TCCCCAGGCCTTACAAGATGATTAT | AAACACTCCCACGTCGCACTAAG | Choi et al 2004 |
| TUP | DX922526 DX922468 | 1 | Mnl I | CAPS | 420 + 200 | 285 + 135 + 200 | GAATGGGATGCTATGGGAAGTG | TGGATCAGTGGCACCATCTTTAT | Choi et al 2004 |
| UDPGD |  | 7 | Mnl I | CAPS | 230 + 1120 | 1350 | CAAAAGCGTTTCATCACTCATCTCT | ATCGTCAAGGCCAGGTTCATAG | Choi et al 2004 |
| UNK27 | DX922529 DX922469 | 4 | Apo I | CAPS | 350 | 250 + 100 | GGCTTCATCGGTTCTCATCTCTGCGA | TGTAATCAGCAGGAGTACAAATTGCAGCCA | Choi et al 2004 |
| UNK3 |  | 4 | Mbo II | CAPS | 80 + 390 | 470 | CACCGGAAATTCAACAGCAAC | GACCTAGGCAACACAACTCCATTA | Choi et al 2004 |
| UNK7 |  | 5 | Xho I | CAPS | 1420 | 1250 + 170 | AAAAAGCAGCAAGAGAAATGTCAAT | GAGAATCTTTCTCCATCGTATCTTACTT | Choi et al 2004 |
